# Topical application of ALA and ALA hexyl ester on a subcutaneous murine mammary adenocarcinoma: tissue distribution

**DOI:** 10.1038/sj.bjc.6600665

**Published:** 2003-02-10

**Authors:** C Perotti, A Casas, H Fukuda, P Sacca, A Batlle

**Affiliations:** 1 Centro de Investigaciones sobre Porfirinas y Porfirias (CIPYP), CONICET and Department of Biochemistry, Facultad de Ciencias Exactas y Naturales, University of Buenos Aires, Argentina

**Keywords:** photodynamic therapy (PDT), aminolevulinic acid (ALA), ALA derivatives (ALA hexyl ester)

## Abstract

Although 5-aminolevulinic acid (ALA)-based photodynamic therapy (PDT) has proven to be clinically beneficial for the treatment of certain cancers, including a variety of skin cancers, optimal tissue localisation still remains a problem. An approach to improve the bioavailability of protoporphyrin IX (PpIX) is the use of ALA derivatives instead of ALA. In this work, we employed a subcutaneous murine mammary adenocarcinoma to study the tissue distribution pattern of the ALA hexyl ester (He-ALA) in comparison with ALA after their topical application in different vehicles. He-ALA induced porphyrin synthesis in the skin overlying the tumour (SOT), but it did not reach the tumour tissue as efficiently. Only 5 h after He-ALA lotion application, tumour porphyrin levels surpassed control values. He-ALA delivered in cream induced a substantially lower porphyrin synthesis in SOT, reinforcing the importance of the vehicle in the use of topical PDT. Porphyrin levels in internal organs remained almost within control values when He-ALA was employed. The addition of DMSO to ALA formulation slightly increased tumour and SOT porphyrin biosynthesis, but it did not when added to He-ALA lotion.

Photodynamic therapy (PDT) of cancer is based on the administration of a photosensitising compound with tumour-localising properties, and subsequent irradiation with light of an appropriate wavelength leading to selective damage to the treated tissue (Henderson and Dougherty, 1992). The administration of 5-aminolevulinic acid (ALA) to cells and tissues results in the production of protoporphyrin IX (PpIX), through the haem biosynthetic pathway, which can be clinically used as a photosensitiser for both photodetection and also as a good alternative of cancer PDT (Kennedy *et al*, 1990,^1996^; Peng *et al*, 1997).

Although ALA-based PDT has proven to be clinically beneficial for the treatment of certain cancers including a variety of skin cancers, optimal tissue localisation is still a problem to be solved. As ALA is a small molecule, it can penetrate through the skin and into tumours after topical application. However, the clinical use of PDT with ALA-induced PpIX is limited by different factors, such as ALA diffusion through biological barriers, localisation of PpIX, availability of oxygen and rapid photobleaching of PpIX (Van der Akker *et al*, 2000a).

Since the skin and very likely the *stratum corneum* are the main biological barriers limiting ALA diffusion, high ALA doses have to be applied to produce sufficient PpIX levels suitable for PDT (Cairnduff *et al*, 1994; Peng *et al*, 1995).

An approach to improve the bioavailability of PpIX is the use of ALA derivatives instead of ALA as precursors for PpIX synthesis. ALA esters, which are more lipophilic than ALA itself, are expected to enhance its uptake, penetration and distribution. Several ALA esters have been synthesised and tested *in vitro* in different cell lines and it has been found that long-chained ALA esters were more efficient than short-chained ones at improving PpIX production in cells (Kloek and Beijersbergen van Henegouwen, 1996; Gaullier *et al*, 1997; Casas *et al*, 2001a).

5-Aminolevulinic acid hexyl ester (He-ALA) has proven to be successful in inducing PpIX production and distribution in human and porcine bladder mucosa *ex vivo* (Marti *et al*, 1999) and in patients (Lange *et al*, 1999).

On the other hand, topical use of ALA esters was not found to be better than ALA at increasing PpIX production, yet a surprisingly high photosensitiser selectivity was achieved (Peng *et al*, 1996; Casas *et al*, 2001b; Moan *et al*, 2001).

Another approach to optimise ALA penetration is the use of enhancers. The results of experiments with penetration enhancers and tape stripping indicate that the *stratum corneum* acts as a barrier against ALA and He-ALA. The use of enhancers like azacycloalkane derivatives augments PpIX production, especially in the case of He-ALA (Van der Akker *et al*, 2000a).

Dimethylsulphoxide (DMSO), which also acts as a penetration enhancer, among other amphiphilic molecules penetrates skin well, probably because the electron-rich part of the molecule has affinity for the aqueous layers of the *stratum corneum* while the electron-poor portion prefers the lipid region (Wiechers, 1989).

It has been shown that DMSO can increase the penetration of various chemical agents through human skin (Stoughton and Fritdch, 1964), although the mechanism is not yet known. It has been reported that DMSO can improve the accumulation of PpIX in cell lines (Malik *et al*, 1995). Employing ALA plus DMSO *in vivo* in a transplantable murine adenocarcinoma, Casas *et al* (2000) have found a higher porphyrin accumulation in the skin overlying the tumour tissue and in the first 2 mm of tumour.

The aim of this work was to improve the clinical application of ALA PDT by studying the tissue distribution pattern of He-ALA compared to ALA after their topical application in two different vehicles in a subcutaneous murine mammary adenocarcinoma implanted in mice. The effect of adding DMSO to the formulations of both compounds was also evaluated.

## MATERIALS AND METHODS

### Animals

Male BALB/c mice 12 weeks old, weighing 20–25 g, were used. They were provided with food (Purina 3, Molinos Río de la Plata) and water *ad libitum*. A suspension of 1.65×10^5^ cells of the LM2 cell line (Galli *et al*, 2000) derived from the murine mammary adenocarcinoma M2 (Instituto Roffo, Buenos Aires) was subcutaneously injected on the flanks of male BALB/c mice. Experiments were performed at approximately day 20 after implantation. Tumours of the same uniform size were employed (1 cm diameter). Animals received human care and were treated in accordance with guidelines established by the Animal Care and Use Committee of the Argentine Association of Specialists in Laboratory Animals (AADEALC), in full accord with the UK Guidelines for the Welfare of Animals in Experimental Neoplasia (Workman *et al*, 1998).

### Drugs

ALA was purchased from Sigma Chemical Co., St Louis, MO, USA. ALA hexyl ester (He-ALA) was synthesised according to the method previously described in Casas *et al* (1999a). All other chemicals were of analytical grade.

### ALA administration

The hydrochloric acid (HCl) salt of ALA and He-ALA were dissolved in saline in a final volume of 0.2 ml immediately before use (lotion). The addition of 5% DMSO to this saline lotion constituted the lotion/DMSO formulation. For the cream preparations, ALA and He-ALA were freshly dissolved in 50 mg of an oil in water emulsion cream (Genargen, Argentina).

Both ALA formulations were applied on the tumour, having shaved the hair and rubbing with a smooth paintbrush for a period of 5 min, time at which no vestiges of either cream or lotion were visible. Before any application, mice received mild anaesthesia (Fentanyl and Diazepam). All formulations were applied under occlusive dressing to avoid distribution of the compounds.

### Tissue porphyrin extraction

After ALA or He-ALA topical application, animals were killed. Before killing mice were injected with heparin (0.15 ml, 1000 UI), and after killing they were perfused with 200 ml of sterile saline. The tissue samples were homogenised in a 4 : 1 solution of ethyl acetate : glacial acetic acid mixture according to Batlle (1997). Briefly, the mixtures were centrifuged for 30 min at 3000 **g**, and the supernatants were added with an equal volume of 5% HCl. Extraction with HCl was repeated until there was no detectable fluorescence in the organic layer. The aqueous fraction was used for the determination of porphyrins. For fluorometric determination, a Shimadzu RF-510 spectrofluorometer was used, with an emission wavelength of 604 nm and an excitation wavelength of 406 nm, employing PpIX reference standard.

### Statistical analysis

The unpaired *t*-test was used to establish the significance of differences between groups. Differences were considered to be statistically significant when *P*<0.05. Three mice per group were employed.

## RESULTS

### Dose course of porphyrin levels after topical application of ALA or He-ALA

**Figure 1:**
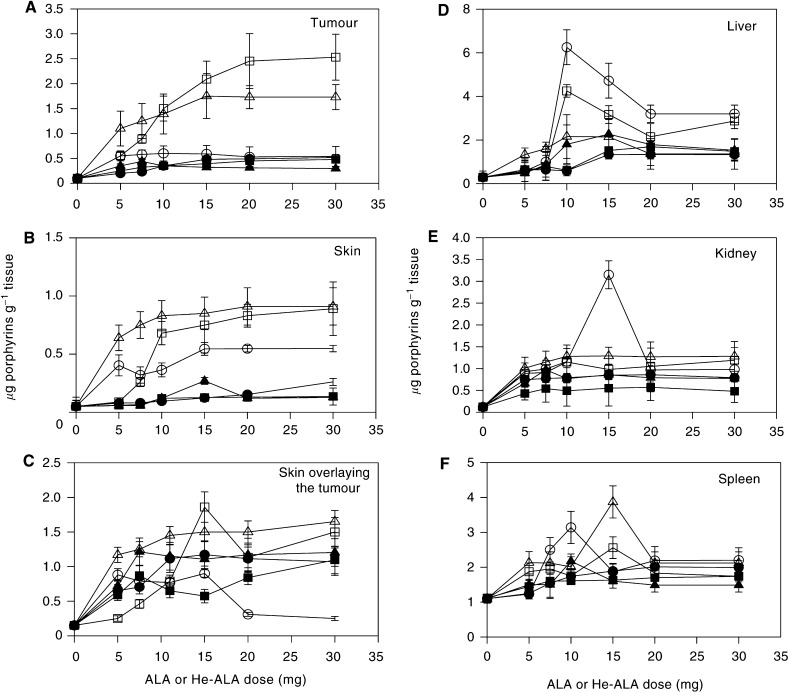
Porphyrin accumulation after topical application on the tumour and skin of various quantities of ALA or He-ALA. Different amounts of ALA cream (○), ALA lotion (▵), ALA lotion/DMSO (□), He-ALA cream (•), He-ALA lotion (▴) and He-ALA lotion/DMSO (□) were applied on the tumour. After 3 h, tumour (**A**), skin (**B**), skin overlying the tumour (**C**), liver (**D**), kidney (**E**) and spleen (**F**) were excised and porphyrins were extracted as detailed in the Materials and Methods. Each data point represents the average of three determinations. Error bars show standard deviations.

The dose course of porphyrin levels in tumour, skin and peritumoural skin is shown in Figure 1A–C

. In tumour, after application of ALA cream or lotion, porphyrin accumulation increased with the amount of ALA applied, reaching a plateau at 5 and 15 mg ALA, respectively. Highest porphyrin levels were found when using ALA lotion with a peak of 1.75±0.45 *μ*g porphyrin g^−1^ tissue at 15 mg ALA. In general, employing the ALA lotion, values 3 times higher than either ALA cream or He-ALA were obtained. When using He-ALA cream or lotion, porphyrin levels do not increase significantly over control values.

The addition of 10% DMSO to ALA lotion increases the amount of porphyrins formed in tumour from 15 mg ALA onwards, although values were not significantly much higher. When employing 20 mg ALA lotion or ALA lotion plus DMSO, 1.75±0.25 and 2.5±0.5 *μ*g porphyrin g^−1^ tumour, respectively, were accumulated. Instead, addition of DMSO to He-ALA lotion did not enhance the amount of synthesised porphyrins.

In normal skin, ALA lotion also induced the synthesis of the highest levels of porphyrin, reaching a plateau of 0.83±0.13 *μ*g g^−1^ at 10 mg ALA. Addition of DMSO to ALA lotion equalled lotion-alone values within the 10–15 mg ALA range, without increasing further porphyrin levels. Again, porphyrins induced by He-ALA in either lotion or cream were within control values.

In the skin overlying the tumour (SOT), plateaus were rapidly reached within the 5–7.5 mg range for both ALA and He-ALA, and porphyrin values were alike for both compounds, independent of the vehicle. ALA cream concentration higher than 15 mg induced significantly lower porphyrin levels compared to those reached at the plateau (0.29±0.04, *P*<0.05). Addition of DMSO to ALA lotion showed a sharp peak at 15 mg ALA (1.8±0.4 *μ*g porphyrins g^−1^ tissue), but no further increase at other points as compared to ALA lotion alone. Addition of DMSO to He-ALA lotion did not enhance porphyrin synthesis but reduced it.

Figure 1D–F depicts porphyrin accumulation in liver, kidney and spleen. In liver tissue, sharp peaks were obtained with 10 mg ALA in cream and lotion plus DMSO (6.2±0.85 and 4.1±0.25 *μ*g porphyrins g^−1^ tissue, respectively). Slight increases of porphyrin levels were found after topical application of He-ALA and ALA lotion, although they were not significantly different from the control.

In kidney, maximum porphyrin accumulation was reached with 15 mg ALA cream (3.2±0.3 *μ*g porphyrins g^−1^ tissue). After application of ALA lotion alone or plus DMSO, values doubled controls. After He-ALA treatment porphyrins did not increase significantly over the control in the range of doses studied, independent of the vehicle.

In spleen, the highest levels of porphyrins were accumulated after applying 10 mg ALA cream and 15 mg ALA lotion (3.2±0.5 and 3.9±0.5 *μ*g porphyrin g^−1^ tissue, respectively). Again, porphyrins were slightly increased over the control after He-ALA treatment without significant changes in the range of doses studied. Addition of DMSO to ALA did not enhance porphyrin formation.

### Tissue distribution of porphyrins after ALA or He-ALA topical application

The pattern of porphyrin accumulation in lung, heart, ear, gut, bladder and brain after application of different amounts of ALA in cream or lotion is shown in Table 1

**Table 1:** Tissue distribution of porphyrins after application of different amounts of ALA cream

	**Lung**	**Heart**	**Ear**	**Gut**	**Bladder**	**Brain**
Control	0.49±0.08	0.29±0.03	0.15±0.05	0.37±0.07	0.36±0.03	0.11±0.01
7.5 mg ALA	0.53±0.07	0.65±0.06	0.20±0.03	1.53±0.23	0.51±0.07	0.11±0.05
15 mg ALA	0.60±0.09	1.89±0.21	0.21±0.05	1.79±0.23	0.63±0.03	0.13±0.01
20 mg ALA	0.43±0.06	2.53±0.15	0.11±0.05	1.83±0.26	0.58±0.07	0.18±0.02
30 mg ALA	0.50±0.07	2.83±0.41	0.20±0.05	2.15±0.31	0.40±0.06	0.17±0.02

Different amounts of ala cream were topically applied on the tumour. Mice were killed 3 h after drug administration and porphyrins (*μ*g g^−1^ tissue) were determined spectrophotometrically.

and 2

**Table 2:** Tissue distribution of porphyrins after application of different amounts of ALA in lotion

	**Lung**	**Heart**	**Ear**	**Gut**	**Bladder**	**Brain**
Control	0.49±0.08	0.29±0.03	0.15±0.05	0.37±0.07	0.36±0.03	0.11±0.05
7.5 mg ALA	0.28±0.05	1.43±0.03	0.58±0.06	1.19±0.03	0.40±0.04	0.15±0.02
15 mg ALA	0.39±0.05	1.53±0.21	0.82±0.07	1.41±0.12	0.53±0.07	0.17±0.03
20 mg ALA	0.45±0.03	1.91±0.41	1.24±0.10	1.43±0.11	0.93±0.06	0.15±0.03
30 mg ALA	0.52±0.04	2.13±0.33	1.45±0.34	1.97±0.25	0.88±0.09	0.20±0.04

Different amounts of ALA in lotion were topically applied on the tumour. Mice were killed 3 h after drug administration and porphyrins (*μ*g g^−1^ tissue) were determined spectrophotometrically.

, respectively. When using both ALA formulations, heart and gut showed a marked increase in porphyrin levels. In addition to these tissues, some enhanced porphyrin accumulation in a dose-dependent way after ALA lotion application was found in ear and bladder. He-ALA cream increases basal porphyrin levels in a dose-dependent way in the heart (control 0.29±0.03 *vs* 30 mg He-ALA 0.57±0.05 *μ*g porphyrin g^−1^ tissue, *P*<0.05) and bladder (control 0.36±0.03 *vs* 30 mg He-ALA 0.83±0.08 *μ*g porphyrin g^−1^ tissue, *P*<0.05), while the other tissues remain within control values. On the other hand, He-ALA lotion did not induce any change in basal porphyrin levels for all the tissues studied (data not shown).

### Time course of porphyrin levels after ALA or He-ALA topical application

**Figure 2:**
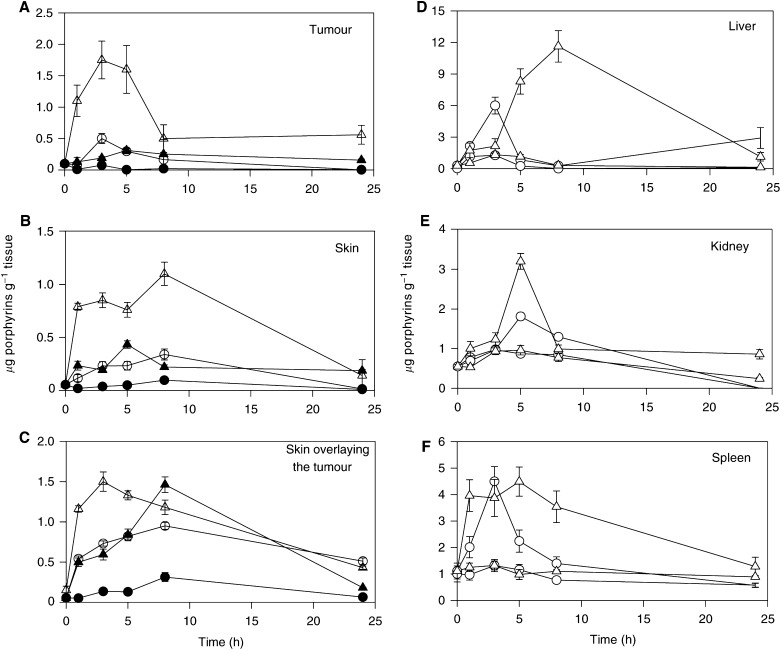
Concentration of ALA and He-ALA-induced porphyrins as a function of time after topical application of ALA and He-ALA in cream or lotion. At different times after tumour topical application of 10 mg ALA in cream (○), 15 mg ALA lotion (▵), 15 mg He-ALA cream (•) and 15 mg He-ALA lotion (▴), tissues were excised and porphyrins were extracted as detailed in the Materials and Methods section (tumour (**A**), skin (**B**), skin overlying tumour (**C**), liver (**D**), kidney (**E**) and spleen (**F**)). Each data point represents the average of three determinations. Error bars show standard deviations.

The time course of porphyrin levels after topical application of ALA or He-ALA cream and lotion on tumour, skin and SOT is shown in Figure 2A–C

. Maximal accumulation was found in tumour 3 h after ALA application. He-ALA lotion induced an increase slightly above control at 5 h (very significant), but He-ALA cream did not produce porphyrin synthesis.

In normal skin, ALA in both preparations induced a peak of porphyrins at 8 h. When using He-ALA in lotion, a defined peak was observed at 5 h but none in its cream formulation.

SOT exhibits a peak 3 h after application of ALA lotion, whereas ALA cream and both He-ALA formulations induced peaks at 8 h. In this tissue, levels obtained with He-ALA lotion were high and equalled those obtained with ALA lotion.

In Figure 2D–F, liver, kidney and spleen time courses are depicted. In these three tissues, ALA in either formulation induced different peak times; instead porphyrin accumulation from He-ALA did not change from basal porphyrin levels in any tissue.

## DISCUSSION

The kinetics of ALA in lotion and cream formulations have already been discussed in a previous work (Casas *et al*, 1999b). Here we will focus on the differences between ALA and He-ALA topically applied, also in lotion and cream vehicles.

From these results, we can conclude that He-ALA induces porphyrin synthesis in the skin overlying the tumour but it cannot reach the tumour tissue as efficiently. Only 5 h after He-ALA lotion application, tumour porphyrin levels surpass control values. Porphyrins formed from He-ALA were more above controls in remote skin tissue than in tumour tissue. Porphyrin concentration in internal organs remained within control values when He-ALA was employed.

Moreover, even in SOT, although He-ALA lotion-induced synthesis equals ALA lotion, the amount of the ester needed is 1.5 higher than that of ALA. He-ALA delivered in cream induced a substantially lower porphyrin synthesis than in saline, reinforcing the importance of the vehicle in the use of topical PDT.

Moan *et al* (2001) studied the PpIX formation after topical application of ALA, ALA methyl ester and He-ALA on normal skin and on skin overlying a subcutaneous human colon adenocarcinoma. By means of an *in vivo* fluorescence signal from SOT measurements, they found that ALA induced the same levels of PpIX as compared to He-ALA applied in a 20% cream formulation, but in remote skin sites no fluorescence was detected. In our work, we have also found that in SOT porphyrin levels induced by He-ALA are equal to those obtained from ALA, and by means of porphyrin extraction we have demonstrated that He-ALA does not induce a significant amount of photosensitiser in the subcutaneous tumour itself.

In accordance with our findings, Van den Akker *et al* (2000b) demonstrated that the *stratum corneum* was the main barrier for He-ALA penetration. They found that the ALA ester did not induce higher PpIX production in normal mouse skin as compared to ALA. Employing a cream formulation and tape stripping of the *stratum corneum*, they demonstrated that this was the layer limiting and lowering PpIX production.

Also Van den Akker *et al* (2000b), using hairless mice, showed that ALA pentyl ester produces only slightly more PpIX than ALA in UVB-induced (pre)cancerous skin lesions, while in normal skin, porphyrin levels were equal from both ALA compounds. They found higher PpIX levels in the *stratum corneum* of lesional skin, but not in the dysplastic layer of the epidermis, showing that this ester also poorly diffuses as compared to ALA. These lesions consisted of actinic and seborrhoeic keratosis and squamous cell carcinomas.

However, in a previous work (Casas *et al*, 2001b) we have analysed porphyrin formation in chemically induced squamous papillomas, after topical application of ALA and He-ALA in cream and lotion formulations. Not only did we find porphyrin synthesis from ALA in these highly keratinised papillomas, but we also found porphyrin accumulation in internal organs, showing that the ALA-hexyl ester does reach the dermis in this tumour model. Moreover, after applying He-ALA in the normal skin of nontumour-bearing mice, we also found the same pattern in internal organs. Even in these hyperkeratotic papillomas, this molecule diffuses freely. We could predict that He-ALA may be highly useful, as it has already been proven with ALA methyl ester (Fritsch *et al*, 1998) in the treatment of actinic keratosis and verruca vulgaris.

In SOT, He-ALA peaks are delayed as compared to ALA lotion, and this can be explained either by considering the additional step of esterase breakage of the alcohol chain, difficulties in crossing the *stratum corneum*. The fact that porphyrin biosynthesis induced from ALA cream is also delayed, favours the latter hypothesis. Moreover, culturing different tissue explants (tumour, normal skin, SOT, liver, kidney and spleen) exposed to ALA or He-ALA, we found that porphyrin levels from either compounds were similar, supporting the proposal that there are no differences on esterase activity for any tissue (Perotti *et al*, 2002).

The attempt to raise tumour porphyrin biosynthesis from He-ALA by adding DMSO failed; on the contrary, the values obtained were even lower. However, Van der Akker *et al* (2000a) found that penetration enhancers like azacycloalkane derivatives augment PpIX production from He-ALA. These differences may be ascribed to different mechanisms of action for both compounds. Penetration enhancers such as DMSO exert a direct influence on the aqueous regions between the polar lipid head groups of the bilayer, altering the solubilising ability of this site, thereby promoting partitioning into the skin. On the other hand, the nonpolar enhancer Azone® exerts its action on the hydrophobic tails of the bilayer by upsetting their packing and increasing their fluidity (Wiechers, 1989).

In conclusion, according to these results and those from Casas *et al* (2001b) when compared to ALA, only superficial skin lesions may be successfully treated with topical He-ALA PDT with the advantage of tumour selectivity; instead malignancies such as chest wall progression of breast cancer will probably fail this treatment.
